# The role of hypothyroidism in the resilience against depressive episodes during covid pandemic in a cohort of old adults

**DOI:** 10.3389/fpsyt.2026.1603347

**Published:** 2026-03-03

**Authors:** Alessandra Perra, Massimo Tusconi, Elisa Cantone, Michela Atzeni, Enzo Tramontano, Stefano Lorrai, Fabrizio Bert, Antonio E. Nardi, Mauro Giovanni Carta, Giulia Cossu

**Affiliations:** 1 Universidad Popular del Cesar, Valledupar, Colombia; 2 Center for Liaison Psychiatry and Psychosomatics, University Hospital of Cagliari, Cagliari, Italy; 3 Department of Life and Environmental Sciences, University of Cagliari, Cagliari, Italy; 4 Department of Public Health Sciences, University of Turin, Turin, Italy; 5 Institute of Psychiatry, Federal University of Rio de Janeiro, Rio de Janeiro, Brazil; 6 Department of Medical Sciences and Public Health, University of Cagliari, Cagliari, Italy

**Keywords:** COVID-19 lockdown, depressive episodes, hypothyroidism, older adults, quality of life, resilience, social rhythms

## Abstract

**Background:**

Hypothyroidism is associated with major depressive disorder and treatment-resistant depression. However, thyroid function tests at rest may not fully capture the thyroid’s stress response, possibly explaining its role in depression vulnerability. This study aimed to assess whether Hypothyroidism affected resilience to depressive episodes in older adults during the COVID-19 lockdown.

**Methods:**

We analyzed a cohort from a randomized controlled trial on active aging conducted a year prior. Participants (aged 65+) were reassessed during the lockdown (March-April 2020). Depression was measured using the PHQ-9, quality of life with the SF-12, and social rhythms with the Brief Social Rhythms Scale. Comparisons between individuals with and without Hypothyroidism were made using Fisher’s exact test and two-way ANOVA.

**Results:**

Of 93 participants, 15 had Hypothyroidism, predominantly female (80% vs. 48.7%, p=0.026). Pre-lockdown depressive episodes were similar between groups (20% vs. 16.67%, p=0.432). During lockdown, depressive episodes decreased in euthyroid participants but increased significantly in those with Hypothyroidism (7.69% vs. 33.3%, p=0.015). Quality of life worsened more in the hypothyroid group (time × group interaction, p=0.005). Social rhythm dysregulation decreased in both groups but remained higher in hypothyroid individuals.

**Conclusion:**

Older adults generally displayed resilience during the pandemic. However, those with Hypothyroidism experienced increased depressive episodes and worsened quality of life, suggesting reduced stress adaptability despite hormone replacement therapy. This consideration highlights the potential need for interventions addressing both biological and psychosocial factors in managing depression risk in hypothyroid individuals.

## Background

Hypothyroidism, characterized by reduced thyroid hormone, is closely associated with major depressive disorder. Studies have shown that Hypothyroidism can trigger affective disorders (1–4) and approximately 1% to 4% of people with depression have overt Hypothyroidism, while a variable percentage between 4% and 40% may have subclinical Hypothyroidism (4–7). Although there is an association between Hypothyroidism and depression, most people with depressive disorders have normal thyroid function (8–10). However, due to the overlap of symptom, namely fatigue and difficulty concentrating, it was suggested to assess thyroid function in people searching for care for depressive symptoms, especially when signs suggestive of thyroid dysfunction are present (11, 12). The association between Hypothyroidism and chronicity, risk of relapse and treatment-resistance in depressive disorders was also found (3, 12, 13). In some cases, thyroid hormone replacement therapy may alleviate depressive symptoms in patients with Hypothyroidism. However, the response to treatment can vary, and normalizing hormone levels does not always lead to complete remission of depressive symptoms, suggesting the possible coexistence of a primary depressive disorder (14). Scientific literature supports the hypothesis that while standard thyroid function tests often assess hormone levels at rest, they may not fully capture the thyroid’s responsiveness under stress, particularly in individuals with a history of depressive episodes. This could explain the observed association between depressive disorders and Hypothyroidism, even when treatment has normalized basal hormone levels (15). Research indicates that minor fluctuations in thyroid hormone levels, even within the normal range, can significantly impact brain function in depressed patients. The limbic system, which is involved in mood regulation and widely expresses thyroid hormone receptors, plays a role in this process. These subtle hormonal changes might not be detected during routine thyroid assessments conducted at rest (15, 16). People with depression and normal thyroid hormone function, when hormone levels were assessed at regular scheduled times as routinely, may not be sufficient to respond to stress in vulnerable conditions as depressive status may led brain sensitivity. So standard thyroid function tests may not fully capture the dynamic relationship between thyroid activity and stress, particularly in individuals with a history of depression (17). As known sustained stress responses can lead to elevated cortisol levels, which in turn affect the synthesis, secretion, metabolism and fluctuation and rhythms of thyroid hormones, increasing the risk of an hypotheses of a sub-sub clinical hypothyroidism in depressive disorders, i.e., a condition in which a normal level of hormones and TSH at rest does not face stress (17).

An alternative hypothesis could be that vulnerability in depressive disorder, with hormone levels within the normal range due to pharmacological control, might also be caused by potential anti-thyroid autoimmunity (18). However, in this case there is the confounding factor due to the stronger association between bipolar disorder and thyroid autoimmunity (19). Since much of the data comes from epidemiological surveys that use screening tools to identify major depressive disorder, there is a potential limitation. These tools do not diagnose major depressive disorder itself (which requires a lifetime diagnosis) but rather detect depressive episodes. Since depressive episodes are also common in bipolar disorders, this may introduce a bias when studying the association with thyroid autoimmunity (20).

In this framework, it is interesting to explore how individuals with Hypothyroidism respond to specific stressors compared to those without Hypothyroidism. Our research team conducted a study on a cohort of elderly individuals who had participated in an active aging intervention a year earlier. This cohort was then observed during the COVID-19 lockdown, which provided an opportunity for a follow-up assessment (21). Remarkably, the study revealed an unexpectedly strong resilience among these older adults in coping with the risk of depression during the pandemic (22), a finding that was later supported by similar studies (23–26).

One key factor contributing to this resilience was the regularity of social rhythms, which had been assessed during the initial intervention a year prior (27). “Resilience” is defined as an individual’s ability to maintain psychological balance in the face of stress (28), thus reducing the likelihood of developing mental health issues. In the context of active aging, the regularity of social rhythms serves as a specific mechanism of resilience, offering protection against depression (22, 25). In fact, a follow-up study from a randomized controlled trial (RCT) highlighted that good regulation of social rhythms one year before the pandemic was associated with a lower risk of depression during the lockdown (22). This suggests that the stability of daily routines plays a crucial role in managing stress, acting as an important resilience factor.

Understanding these risk and protective factors is fundamental for guiding intervention efforts, as it sheds light on the underlying mechanisms of resilience (1, 29, 30).

The aim of this study was to verify whether the presence of Hypothyroidism was associated with a different level of resilience in old adult during the pandemic.

## Methods

### Design and setting

The study is based on a cohort from a randomized controlled trial on active aging conducted over multiple time points. The one-year follow-up evaluation coincidentally took place during the COVID-19 lockdown.

The sample in this study included only individuals who had undergone an initial assessment at T0, were re-evaluated at the end of the RCT in both study arms (31), and were subsequently assessed during the final evaluation conducted during the lockdown, which, along with T0, will be the focus of the present analyses. Specifically, in the analyzed sample, outcome measures were assessed and compared in March-April 2019 and again in March-April 2020.

### Study sample and recruitment

The study included individuals over the age of 64 living at home, with no restrictions on sex. One of the randomized interventions consisted of a low-to-moderate intensity exercise program, while the active control group participated in a series of cultural meetings. Given the moderate nature of the exercise, individuals with non-severe chronic diseases were also eligible to participate, representing a significant portion of the sample. Exclusion criteria were limited to individuals with severe obesity or those deemed unfit for mild-to-moderate physical activity due to serious medical conditions.

Participants located in the metropolitan area of Cagliari and were assessed and enrolled as part of the study at the University Hospital and recruited through the Consultation-Liaison Psychiatry and Psychosomatics Center. The study was advertised to the community via media and public notices, and those interested in participating were required to contact their general practitioners (GPs), who acted as intermediaries with the study organizers. Participants were randomly assigned in a 1:1 ratio using a computer-based, double-blind method with permuted blocks (stratified by age and sex) and blind codes, ensuring pseudonymized identification. This approach maintained anonymity and concealed participants’ group allocation throughout all assessment phases of the study in a blind design.

### Tools

Demographic basic data were collected thorough a specifically developed structured tool. The Patient Health Questionnaire version of 9 items (PHQ9) is a self-administered tool capable to detect the presence of depressive symptoms during the past 2 weeks (32, 33), it was used in the Italian previously validated version (33, 34). Each of the nine items of the questionnaire correspond to the one of the nine depressive “core” symptoms useful for a diagnosis of depressive episode according to the DSM-5 classification (35). The level of the severity in each symptom/item is collected in a Likert scale scoring from 0 (meaning at “not at all”) to 4 (meaning “maximum severity” or “every day”). A score over the cut-off of 4/5 identifies a depressive episode of severity at least mild (32). The internal consistency is Cronbach’s α = 0.89 (32).

The health-related quality of life (H-Qol) into two groups one year before and during the lockdown was measured by the Short-Form Health Survey (SF-12) (36, 37). SF-12 consists of a questionnaire of 12 items aiming to measure how health, into the physical and psychological components. is perceived to impact the daily life of a person. The higher is the score, the better is the perception of the quality of life. Its internal consistency is Cronbach’s α = 0.94 (38).

The functionality and synchronism of the social and behavioral rhythms were assessed through the Italian version (39) of Brief Social Rhythms Scale (BSRS) (40). It is a 10 items tool conceived to measure the synchronism/variability in daily routines in a week inkling a separate evaluation of the workweek and the weekend. Namely the regularity of social interactions, sleep patterns and mealtimes. The is higher the score, the worse is the functioning and regularity of social and individual rhythms. Without a standardized cut-off, we used scores above the mean plus one standard deviation to indicate rhythm dysfunction (27). Its internal consistency is Cronbach’s α value is 0.912 (39).

### Statistical analysis

Statistical analysis was carried out as measure as comparison of the presence in the two groups (with and without Hypothyroidism) of people with depressive episode and with dysregulation rhythms at the entry of the study (one year before Covid-19) and during the lockdown. As they were nominal variables measured on small samples, it was not possible to conduct a multivariate analysis for time and group, the differences in the two groups in the response to “lockdown and pandemic stress” were therefore conducted with the Fisher exact test comparing the differences in the groups before and after with two separate analyses. To analyze whether there was a different response in the two groups of the perception of quality of life (SF-12 score) (37) in relation to the stressful event pandemic lockdown, we conducted a repeated measures analysis of variance (two-way ANOVA) with the factors: Time (pre vs. post stressful event); Group and we verified whether there is a significant interaction between these factors.

### Ethics

The ethics committee of the University Hospital of Cagliari, Italy, approved the final study protocol (October 25th, 2018; registration number PG/2018/15546). Participants have given a written consensus about their participation on the study after having been explained that the data would be collected in an anonymous database according the European data protection laws. The study was carried out according to Helsinki Declaration.

## Results

**Table 1** show the characteristics by sex and age of the two samples at the entry into the study. The two groups have similar average ages but the 15 people with previous Hypothyroidism have a higher percentage of females than the 78 people without Hypothyroidism (80% vs 48.71%, χ2 1df=4.952; P = 0.026; OR (F)=4.21; CI 95% (1.10-16.09). Of the 15 people with previous Hypothyroidism, 12 (80%) suffered from thyroiditis, 3 (20%) underwent thyroidectomy, all are currently undergoing replacement therapy with periodic checks.

**Table 1:** Characteristics of the two samples at the entry into the study.

Variable	Old adults sample one year before covid lockdown N = 93	With hypothyroidism N=15	Without hypothyroidism N=78	Statistics
Females (%)	50 [53.8%]	12 (80%)	38 (48.71%)	χ2 1df=4.952 P=0.026 OR (F)=4.21 CI 95% (1.10-16.09)
Age	73.36±4.97	72.73±4.05	73.48±5.14	ANOVA 1way 1,91 df F=2.84; p=0.595

In the **Table 2** is presented the frequency of depressive episodes (score PHQ9>4) in people with or without Hypothyroidism one year before and during the lockdown of the Covid pandemic. Depressive Episodes were balanced one year before the lockdown, specifically 16.67% in people without against 20% in people with Hypothyroidism (Fisher Exact test p=0.432; OR = 1.70; CI 95% 0.40-7.09). In people without Hypothyroidism, the frequency of depressive episodes decreases during the lockdown, while it increases in people with Hypothyroidism so much that the difference between the two groups becomes statistically significant (7.69% vs 33.3%; Fisher Exact test p=0.015; OR = 5.38, CI 95% 6.00-23.347). The analysis of the comparison between people without Hypothyroidism and different forms of hypothyroidism highlights that in people with previous thyroiditis the frequency of depressive episodes tendentially increases during the lockdown and this brings the difference with people without Hypothyroidism to the limits of statistical significance during the lockdown (7.69% vs 25%; Fisher Exact test p=0.097; OR = 4.00; CI95% 0.85-18.3); in people with Hypothyroidism due to thyroid removal the frequency of depressive episodes remains stable during the lockdown and this brings the difference with people without Hypothyroidism to reach of statistical significance due to the tendentially decreases of depressive episodes in people without Hypothyroidism (7.69% vs 66.67%; Fisher p=0.025; OR = 24.00, CI 95% 1.89-116.8). In the study cohort as a whole (N = 93), the proportion of people with a depressive episode did not change with statistical differences between before and during the lockdown, in fact it went from 16/93 (17.20%) to 11/93 (11.82%) (χ2 = 1.083; p=0.298; OR = 1.54; CI95% =.67-3.54). The frequency of depression is, as expected, higher in females (11 out of 50, 22%) versus males (5 out of 43, 11.6%) but the difference does not reach statistical significance (Fisher p= 0.271, OR Male =0.467, CI 95% 0.14-1.57); a trend of a slightly lower loss of the frequency of depressive episodes is observed in males but a statistically significant difference between the sexes is not reached (18% females vs 4.65%; Fisher Exact test p = 0.060; OR Males =0.220, CI% 050.04-1.08).

**Table 2:** Frequency of depressive episodes (score PHQ9>4) in people with or without Hypothyroidism one year before and during the lockdown of the Covid pandemic.

Group	Without hypothyroidism N = 78 (pivot)	With hypothyroidism N=15	Fisher exact test	With hypothyroidism thyroiditis N=12	Fisher exact test	With hypothyroidism from thyroid removal N=3	Fisher exact test
Depressive Episodes (PHQ9>4) One year before the lockdown	13 (16.67%)	3 (20%)	p=0.432 OR = 1.70 (0.40-7.09)	1 (8.3%)	p=0.683 OR = 0.45 (0.09-3.83)	2 (66.67%)	p=0.089 OR = 9.84 (0.83-18.3)
Depressive Episodes (PHQ9>4) during the lock down	6 (7.69%)	5 (33.3%)	p=0.015 OR = 5.38 (6.00-23.347)	3 (25%)	p=0.097 OR = 4.00 (0.85-18.3)	2 (66.67%)	p=0.025 OR=24.00 (1.89-116.8)

People with high social rhythm dysregulation (SBRS score >mean + 1 standard deviation) one year before lockdown were more frequent among those who had suffered from Hypothyroidism and who were currently undergoing treatment compared to the control group (26.67% vs 12.8%; Fisher Exact test, p=0.432 OR = 1.70, CI 95% 0.40-7.09; see **Table 3**), but the difference did not reach statistical significance. During the lockdown, the frequency decreased in both groups (in an apparently paradoxical way) and in this case the difference between the two groups is at the limit of statistical significance (20% vs 12.5%; Fisher Exact test p=0.099; OR = 3.18, CI95% 0.81-12.37).

**Table 3:** Frequency of people with high dysregulation of social rhythms (score BSRS≥28) in people with or without Hypothyroidism one year before and during the lockdown of the Covid pandemic.

Group	Without hypothyroidism N = 78 (pivot)	With hypothyroidism N=15	Fisher exact test
With high dysregulation of rhythms one year before the lockdown	10 (12.8%)	4 (26.67%)	p=0.432 OR=1.70 (0.40-7.09)
With high dysregulation of rhythms during the lockdown	8 (10.25%)	3 (20%)	p=0.099 OR=3.18 (0.81-12.37)

**Table 4** shows the comparison on the perception of Health-Related Quality of Life (assessed by the score of SF-12) in people with or without Hypothyroidism one year before and during the lockdown of the Covid pandemic. The comparison was carried out with repeated measures analysis of variance (two-way ANOVA) considering time (pre-post), group (with and without Hypothyroidism) and the interaction between time and group. The key emerging findings were: 1) Between-Group: Difference of Statistic significant (p = 0.014), indicating that the two groups differ in mean scores regardless of time; 2) Difference in Time: Statistic significant (p = 0.006), suggesting that the stressful event “covid pandemic” had an impact on mean scores overall; 3) Group × Time interaction: Significant (p = 0.005), indicating that the effect of time is different in the two groups, i.e. the response to the stressful event “covid pandemic” was not the same for both groups but stronger in the hypothyroid group more decreasing quality of life.

**Table 4:** Health Related Quality of Life (score SF-12) in people with or without Hypothyroidism one year before and during the lockdown of the Covid pandemic. Repeated measures analysis of variance (two-way ANOVA) by time and group.

Group	Without Hypothyroidism N = 78 (pivot)		With Hypothyroidism N=15
T0	35.92±3.78		34.73±8.34
During Lock Down	34.63±6.29		30.8±10.91
Repeated measures analysis of variance (two-way ANOVA)	sum_sq	F	P
C(Group)	256.06	6.13	*0.014*
C(Time)	315.94	7.56	*0.006*
C(Group):C(Time)	341.72	8.18	*0.005*
Residual	7601.24		

## Discussion

The results of this work indicate that, faced with the stress represented by the covid pandemic and the consequent lockdown, a cohort of old adults, overall, does not show an increase in depressive disorders or a worsening of the quality of life. A small sub-cohort of people with previous Hypothyroidism currently on replacement treatment appeared instead more vulnerable to stress in terms of an increase in depressive episodes and a worsening of the quality of life. This could be associated with an increase in the dysregulation of social rhythms, but the study does not clarify this possible association. We cannot therefore confirm whether the supposed syndrome of rhythm dysregulation and hyperactivity (DYMERS) which includes areas of vulnerability to stress from normal (41, 42) to pathological (43, 44), may have played a role.

The study context, occurring during a critical phase of the COVID-19 pandemic, offered a unique opportunity to observe stress-related dynamics in a particularly vulnerable population. However, it also underscores the need for additional investigations that can replicate these findings under more controlled and diverse conditions.

Several scientific studies have examined the impact of the COVID-19 pandemic and associated lockdowns on depressive disorders and quality of life among older adults. While some research indicates an increase in depression and a decline in quality of life during the pandemic, other studies (the majority) suggest that older adults have demonstrated resilience in the face of these challenges.​ Increased depression and declined quality of life was found in a study carried in *Belgium* highlighted that the COVID-19 pandemic had a severe impact on the mental health of older adults, indicating a decline in their well-being, depression was strongly related to reported declines in activity level, sleep quality, wellbeing and cognitive functioning (45). ​In England a survey reported significant increases in depression, loneliness, and poor quality of life among older adults during June and July 2020 compared to pre-pandemic levels (46). However, the condition subsequently improved dramatically (47) and the resilience among older adults was confirmed by several other surveys (22–27). Several elements have been identified as potential risk factors for depressive disorders and reduced quality of life among older adults during the pandemic:​ greater exposure to risk of death, older adults faced heightened health risks due to weakened immune systems and a greater prevalence of chronic conditions, contributing to increased anxiety and stress (48); dysregulation of rhythms imposed by lockdown, lockdowns and social distancing measures led to reduced physical activity and disrupted daily routines, which are associated with mood disorders (31, 49); Greater detachment and inability to meet friends and relatives: Social isolation intensified feelings of loneliness among older adults, impacting their mental health and quality of life (50).

Whatever the specific aspects of resilience in the elderly, good thyroid function is a relevant factor. Thyroid hormones play a significant role in the body’s response to stress, primarily through their interactions with the hypothalamic-pituitary-thyroid (HPT) axis and their influence on metabolic processes (51).​ Corticosterone, the end product of hypothalamic-pituitary-adrenal (HPA) axis activation and strongly regulated by stress, plays a role in the regulation of the hypothalamic-pituitary-thyroid (HPT) axis, in fact repeated exposure to acute stress causes a decrease in peripheral thyroid hormone levels in relation to the alteration of cortisol (52).

In this case, however, the sub-cohort identified as at risk consists of individuals whose normal thyroid function is maintained through replacement therapy. The study therefore suggests that the baseline function ensured by replacement therapy may not provide the secretory flexibility needed to accommodate the rapid and continuous adaptations required in response to stress.

It is important to note, however, that our mechanistic interpretation remains speculative, as no direct hormonal measurements were collected in this study. The hypothesis of reduced stress-related thyroidal flexibility should therefore be viewed as a conceptual framework rather than an empirically demonstrated pathway. The present findings are best understood as hypothesis-generating evidence that highlights a potential avenue for future research rather than establishing a causal mechanism.

The study, given the limited size of the sample, is not able to establish whether the presumed role of thyroid autoimmunity as a risk factor for depressive disorders could be hypothetically alternative or synergistic to the possible low flexibility of the thyroid response to stress as a cause of depressive risk (and the consequent lowering of quality of life).

Another important aspect, if confirmed, is that the complexity of the stress response seems to involve in a biunivocal way so-called “strictly biological” factors such as hormone levels with so-called “strictly social” factors such as the sense of threat. This would confirm the need for complex and integrated responses that range from improving pharmacological control with improvement of hormonal response to synergistic interventions of psychosocial support (53, 54).

Although this study is based on an unpredictable event that provided the authors with a unique opportunity to investigate potential mechanisms under exceptional conditions, such as the lockdown, it nonetheless has several clear limitations. First, the small sample size and, consequently, the fact that the study was not originally designed to test the hypotheses explored in this secondary analysis.

However, precisely due to the exceptional nature of this observational perspective, we believe that the findings warrant dissemination, particularly in light of future research that will need to validate and potentially confirm them. Additionally, they serve as a valuable basis for generating hypotheses to guide further investigations.

The most substantial limitation of this study is the small number of participants with hypothyroidism (n = 15), which reflects the natural clinical composition of the original randomized controlled trial cohort rather than a targeted sampling strategy. This subgroup emerged naturally within a pre-existing randomized controlled trial cohort and could not be intentionally expanded or balanced. As a result, the proportion of individuals with hypothyroidism did not approximate that of the euthyroid group, limiting the generalizability of the observed associations. The findings should therefore be interpreted as preliminary and hypothesis-generating. This imbalance markedly restricts the generalizability of our findings and underscores that the observed associations should be interpreted as preliminary. Larger, prospectively designed studies with adequate stratification by thyroid status are warranted to confirm whether the patterns identified here persist in more representative populations. Future research with larger and deliberately stratified samples—ideally with more balanced representation of thyroid conditions—will be essential to confirm and refine these results, particularly given the heightened vulnerability of older adults during the COVID-19 pandemic.

Gender imbalance represents an additional methodological constraint, as women—who carry a higher baseline risk of depressive symptoms—were disproportionately represented in the hypothyroid group. Because the male subgroup was too small to permit a reliable gender-stratified analysis, we could not formally test sex-specific effects. While the descriptive trends did not indicate that gender fully explained the differential vulnerability observed during lockdown, future investigations must incorporate sufficient sample sizes to examine sex–thyroid interactions with adequate statistical power.

A further major limitation is the absence of biochemical data on thyroid function (TSH, free T3, free T4). Because the original randomized controlled trial did not include hormonal assessments, thyroid status at follow-up could only be classified clinically based on previous diagnosis and ongoing replacement therapy. As a consequence, we were unable to explore whether reduced hormonal adaptability to stress, a proposed mechanism linking hypothyroidism to increased vulnerability, was directly involved in the differential response observed during the lockdown. The mechanistic interpretation should therefore be considered speculative. Future studies integrating endocrine biomarkers with psychological and behavioral measures will be crucial to test this hypothesis more rigorously.

More broadly, the study must be interpreted as hypothesis-generating. The absence of biological markers, the limited sample size, and the exploratory nature of subgroup analyses prevent definitive conclusions regarding mechanisms linking hypothyroidism to differential stress vulnerability. Replication in larger cohorts, ideally incorporating endocrine assessments and longitudinal stress biomarkers, will be essential to confirm or refute the patterns observed here.

Finally, the study design and sample size also prevented both the application of multiple comparison corrections— which would disproportionately inflate Type II error in a small exploratory dataset— and meaningful stratification by hypothyroidism etiology. Although descriptive patterns are reported, the thyroidectomy subgroup (n = 3) is too small to support reliable inference. This further underscores the need for adequately powered studies specifically designed to disentangle etiological differences in stress-related vulnerability among individuals with thyroid disease.

Understanding the resilience factors that operate in highly stressful contexts is crucial from both a prevention and rehabilitation perspective, as it allows for the development of interventions that consider the complexity of individual needs (in alignment with the identified risk factors). In particular, preventive and rehabilitative interventions should be designed to strengthen resilience factors.

## Conclusions

Elderly with previous Hypothyroidism currently on replacement therapy seem to have been particularly sensitive to the complex chronic stress caused by the pandemic and the lockdown. Future studies will have to verify these results and if they confirm them, they should establish the role of the poor flexibility of the hormonal response guaranteed by replacement therapy together with the possible role of autoimmunity.

## Data Availability

Due to privacy and ethical issues, data are not publicly available. The data presented in this study are available upon request from the corresponding author.
